# Improving maize LAI estimation by integrating multispectral imagery and digital surface models with ensemble learning

**DOI:** 10.3389/fpls.2026.1844975

**Published:** 2026-06-01

**Authors:** Wenfeng Li, Shu Lou, Jizhong He, Jianghua Zhao, Xi Liu, Guodong Fu, Kun Pan, Tong Li

**Affiliations:** Yunnan International Joint Laboratory of Smart Crop Production, Yunnan Agricultural University, Kunming, China

**Keywords:** DSM, LAI, maize, stacking ensemble learning, UAV remote sensing

## Abstract

To overcome the limitations of single remote-sensing features in estimating maize canopy leaf area index (LAI), this study developed a UAV-based estimation approach by integrating multispectral vegetation indices (VIs) with digital surface model (DSM) features and stacking ensemble learning. Field experiments were conducted in Dehong, Yunnan Province, China, during 2023-2024, and UAV multispectral images and DSM products were acquired for maize grown under three planting-density treatments. Five vegetation indices and three DSM-derived texture/structural features were retained according to their correlation with measured LAI, statistical significance, and complementary spectral or structural information. The VI-based random forest (VI-RF) model achieved an R^2^ of 0.835 and an NRMSE of 9.5%, whereas the DSM-based model showed lower performance (R^2^ = 0.641; NRMSE = 14.2%). Under the same random-forest modeling framework, fusing VIs with DSM features improved the overall model performance to R^2^ = 0.892 and NRMSE = 7.6%, indicating that DSM-derived structural information mainly enhanced the feature representation of maize LAI. Using the same VI-DSM feature set, the stacking model with support vector machine (SVM) as the meta-learner further improved the overall performance to R^2^= 0.930 and NRMSE = 6.3%. The additional gain from stacking was moderate but consistent, whereas feature fusion contributed the dominant improvement. The combined VI-DSM-Stacking workflow improved prediction stability across planting densities, especially under low- and high-density canopy conditions where soil background interference and spectral saturation were more evident. These results demonstrate that integrating spectral and DSM-derived structural information with stacking ensemble learning can improve the accuracy and robustness of UAV-based maize LAI estimation.

## 1 Introduction

Maize, one of the world’s three major staple crops and among the most widely cultivated crops globally, is a cornerstone for ensuring food security. It also serves as a critical source of feed, bioenergy, and industrial raw materials. Therefore, monitoring maize growth status and forecasting yield are essential supports for food security and are of great significance to sustainable agricultural development (Jin et al., 2015). In this context, rapid and accurate monitoring of maize growth is crucial. Leaf area index (LAI) is a key physiological parameter characterizing maize canopy structure (Parker, 2020) and has been extensively investigated. LAI directly reflects the canopy’s capacity to intercept photosynthetically active radiation, as well as transpiration and biomass accumulation efficiency, making it a fundamental indicator for crop growth monitoring, yield prediction, and cultivation management. Accurate LAI monitoring is thus important for optimizing field management and improving maize yield. Conventional LAI measurements rely primarily on manual destructive sampling or instrument-based methods. Although these approaches can achieve high accuracy, they are often labor-intensive and time-consuming, may be destructive to plants, and are difficult to implement for high-frequency monitoring at field scales (Hirooka et al., 2018; Strachan and McCaughey, 1996; Vincent et al., 2017).

In recent years, UAV remote sensing has become an important technical approach for retrieving field-scale crop phenotypic parameters due to its advantages of high spatial resolution, flexible data acquisition, and relatively controllable costs (Yang et al., 2017). Considerable progress has been made in estimating LAI using vegetation indices (VIs) derived from UAV multispectral or hyperspectral imagery (Dong et al., 2019). Previous studies have shown that VIs can, to some extent, capture variations in canopy greenness and biomass (Dong et al., 2019). However, the relationship between LAI and spectral responses does not always remain stable and monotonic. During early growth stages with low canopy cover, mixed soil–vegetation reflectance can reduce the sensitivity of VIs to LAI (Liu et al., 2023a); under high cover or canopy closure conditions, some indices tend to saturate, leading to diminished discrimination at high LAI levels (Gao et al., 2023). Moreover, LAI inherently represents an integrated effect of the three-dimensional canopy structure, whereas relying solely on spectral features often fails to adequately characterize spatial structural differences of the canopy and their density-dependent influences. This limitation can compromise model adaptability and robustness across planting densities and phenological stages (Zou et al., 2024).

To address the limitations of single spectral features, multi-source feature fusion has increasingly been recognized as an important direction for improving LAI estimation accuracy (Zhang et al., 2022; Sun et al., 2023; Du et al., 2024b; Zhao et al., 2025). Recent UAV-based studies have shifted from simple VI-LAI empirical relationships toward feature systems that combine spectral indices, texture descriptors, canopy height or DSM-derived structural variables, and super-resolution or deep-feature representations. For example, UAV-based multi-source fusion has been shown to improve LAI estimation in rice and soybean by combining multispectral, RGB, texture, and structural information (Du et al., 2024b; Zhao et al., 2025). In maize, recent studies have also suggested that integrating spectral and structural features can improve LAI estimation under heterogeneous canopy conditions (Chen et al., 2025). However, many available studies still focus on RGB/multispectral texture or canopy height alone, and the specific value of DSM-derived texture features for maize LAI estimation under different planting-density conditions remains insufficiently clarified. Digital surface models (DSMs) can provide geometric information related to canopy roughness, spatial continuity, and height variation. Therefore, fusing DSM-derived features with VIs is expected to provide complementary information under two challenging conditions: soil background interference in sparse canopies and VI saturation in dense canopies.

Beyond feature-level fusion, algorithm-level ensemble learning can further enhance LAI retrieval when multi-source predictors introduce nonlinear relationships, feature redundancy, and scale differences. Stacking ensemble learning is particularly relevant in this context because it does not rely on a single learner to represent the entire VI-DSM-LAI relationship. Instead, base learners with different fitting mechanisms can capture complementary response patterns: tree-based learners can model nonlinear interactions and threshold effects, boosting learners can improve weak signal extraction, and distance-based learners can exploit local sample similarity. The meta-learner then combines these heterogeneous outputs and may reduce the bias or variance associated with any single model. Recent UAV-based crop studies have reported that stacking models can improve the robustness of growth-parameter estimation under multi-feature settings (Du et al., 2024a; Zhao et al., 2024; Zhang et al., 2024; Wang et al., 2026). In the present study, stacking was therefore used as an additional modeling strategy on top of VI-DSM feature fusion. The interpretation was revised to distinguish the dominant contribution of feature fusion from the further but more moderate contribution of stacking under the same fused-feature set.

In this study, field experiments were conducted in Dehong Prefecture, Yunnan Province, China, under different maize planting densities. UAV-based multispectral imagery and DSM data were used to derive vegetation indices (VIs) and DSM texture features, which were then integrated to construct a fused feature dataset. A stacked ensemble learning framework was further introduced for LAI estimation and comparative analysis. The objectives of this study were to: (1) evaluate the effectiveness of fusing multispectral VIs with DSM texture features for maize LAI prediction; (2) compare the performance of stacking ensembles built with different meta-learners; and (3) quantify the effects of planting density on the accuracy and robustness of multi-source remote-sensing-based LAI prediction models. Ultimately, we aim to establish a reusable data-processing and modeling workflow that provides methodological support for high-accuracy LAI monitoring of maize across varying planting densities.

## 2 Materials and methods

### 2.1 Study area and experimental design

**Figure 1:**
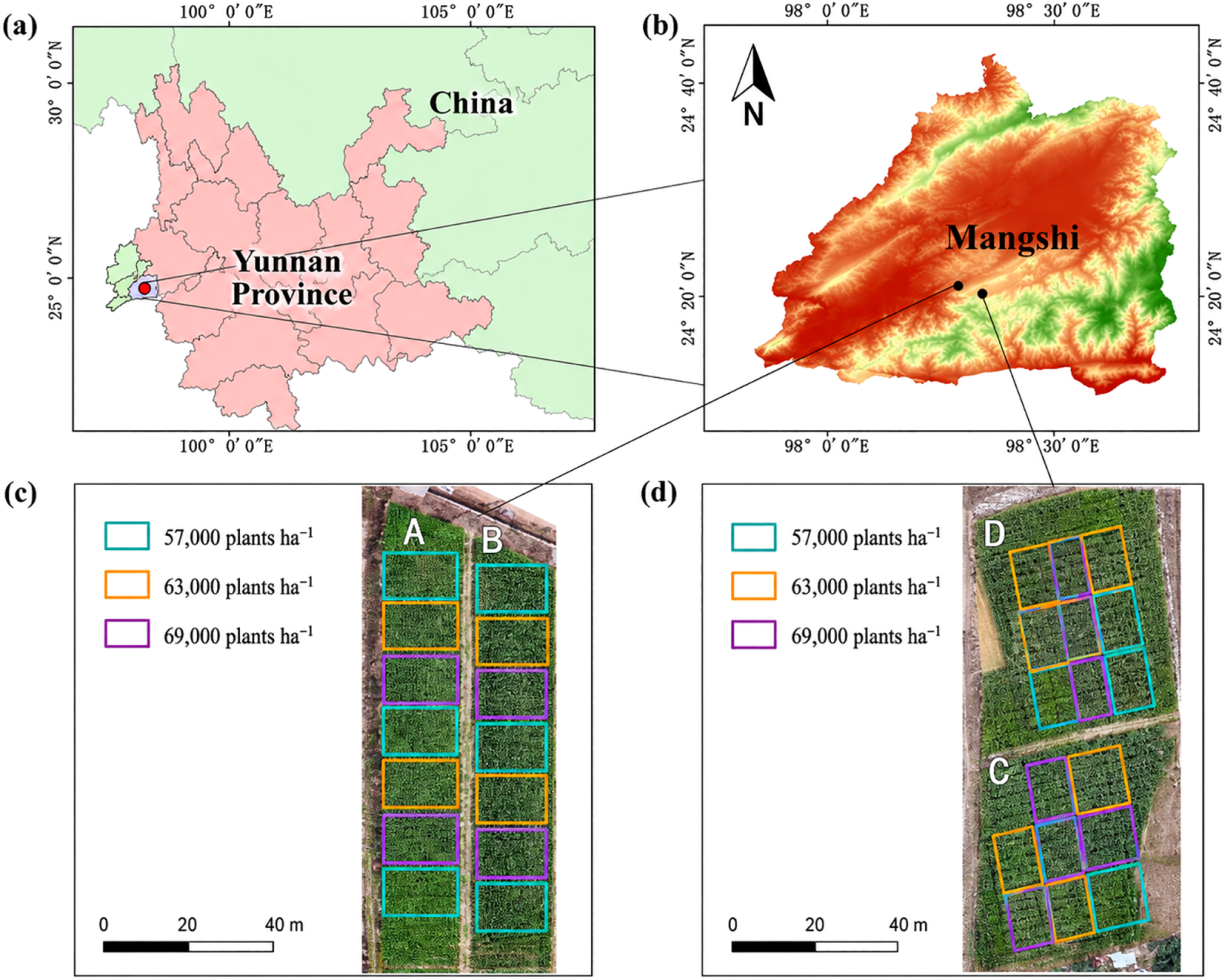
Overview of the experimental area and field layout. **(A)** Location of Yunnan Province in China; **(B)** location of Mangshi, Dehong Prefecture, Yunnan Province; **(C, D)** layouts of the Daxingzhai and Mabozicun experimental fields. Density treatments are indicated by polygon borders: low density (57,000 plants ha−1), medium density (63,000 plants ha−1), and high density (69,000 plants ha−1). Scale bars in **(C, D)** are standardized at rounded values (0, 20, and 40 m). Background orthomosaic color differences do not denote treatment classes unless otherwise indicated in the legend.

This study was conducted from November 2023 to June 2024 in two adjacent sites in Mangshi, Dehong Prefecture, Yunnan Province, China: Daxingzhai (24°26′02″N, 98°35′50″E) and Mabozicun (24°25′48″N, 98°39′55″E) (**Figure 1**). The experimental area is characterized by a South Asian subtropical monsoon climate, with an elevation of approximately 1,200 m. The mean annual air temperature is 19.6 °C, with a recorded maximum of 36.2 °C and a minimum of -0.6 °C. The frost-free period exceeds 300 days. The annual precipitation is 1,638.7 mm, with seasonal totals of 225.0 mm in spring, 1,015.9 mm in summer, 361.9 mm in autumn, and 51.8 mm in winter. Annual sunshine duration is 2,252.9 h (6.2 h/day on average). The soil is sandy loam with a pH of 7.27. Soil organic matter content is 16.9 g/kg, and the available nutrient contents are 72.1 mg/kg for nitrogen, 15.0 mg/kg for phosphorus, and 91.3 mg/kg for potassium. The maize cultivar Beiyu 1521, which exhibits strong disease resistance, was used in the experiment.

The field experiment included three sowing-date batches and three planting-density levels. The three sowing dates were 20 November 2023 (Mabozicun Field A), 10 December 2023 (Mabozicun Field B and Daxingzhai Field C), and 22 January 2024 (Daxingzhai Field D). These sowing-date batches were arranged to broaden the range of canopy development and LAI variation observed during the UAV campaigns, rather than to serve as an independent explanatory variable in the final remote-sensing retrieval model. The primary agronomic treatment evaluated in the model comparison was planting density, with three levels: 57,000, 63,000, and 69,000 plants ha−1 (covering approximately ±9.5% around the conventional density). For all density treatments, row spacing was fixed at 70 cm, while within-row plant spacing was adjusted according to density, i.e., 24.0, 22.7, and 20.7 cm, respectively. The expression ‘nine subplots’ in the original manuscript referred to the layout within each field block, not to the total number of experimental plots. Within each field block, the three density treatments were each replicated three times, yielding nine independent subplots (3 densities × 3 replications). Because the second sowing-date batch was implemented in two adjacent fields to satisfy field-area requirements, the physical field layout included four field blocks. The three replications were independent subplots rather than repeated measurements within a single plot. Adjacent subplots were separated by 1 m buffer strips, and 2 m border rows were maintained around each field. After emergence, pest control was applied four times at 10-day intervals, alternating between lambda-cyhalothrin and fipronil. All other field management practices followed local agronomic standards.

### 2.2 Data acquisition and processing

#### 2.2.1 LAI measurement

LAI was measured using the direct method. In each subplot, three healthy maize plants were randomly selected. For each plant, the length and width of all leaves were measured to calculate the total leaf area per plant. The subplot LAI was represented by the mean leaf area of the three sampled plants. The LAI was calculated as follows:


$$LAI=\frac{1}{A}0.75*\left({\sum_{i=1}^{n}a_{i}*b}_{i}\right)$$

where aij and bij represent the length and maximum width of the j-th leaf of the i-th maize plant, respectively; n denotes the total number of leaves per plant; and A denotes the ground area represented by one plant rather than the projected leaf surface area. Specifically, A was derived from the planting density as A = 10,000/D, where D is the planting density (plants ha−1). Thus, planting density was explicitly incorporated into the LAI calculation. The plot-level LAI was calculated from the mean leaf area of the three sampled plants divided by the corresponding ground area per plant. Field sampling was conducted according to observed phenological stage rather than calendar date alone. The calendar dates (9 March, 22 April, and 18 May 2024) indicate the UAV–ground measurement campaigns corresponding to the seedling, tasseling, and grain-filling sampling windows, respectively. Phenological classification was checked in the field before sampling, and only plants consistent with the target stage were used for the corresponding stage-level dataset. The same number of samples was collected for each planting-density treatment. A total of 81 samples were obtained at the seedling stage, and 108 samples were obtained at both the tasseling and grain-filling stages, yielding 297 plant samples in total.

#### 2.2.2 UAV remote sensing data acquisition and processing

**Figure 2:**
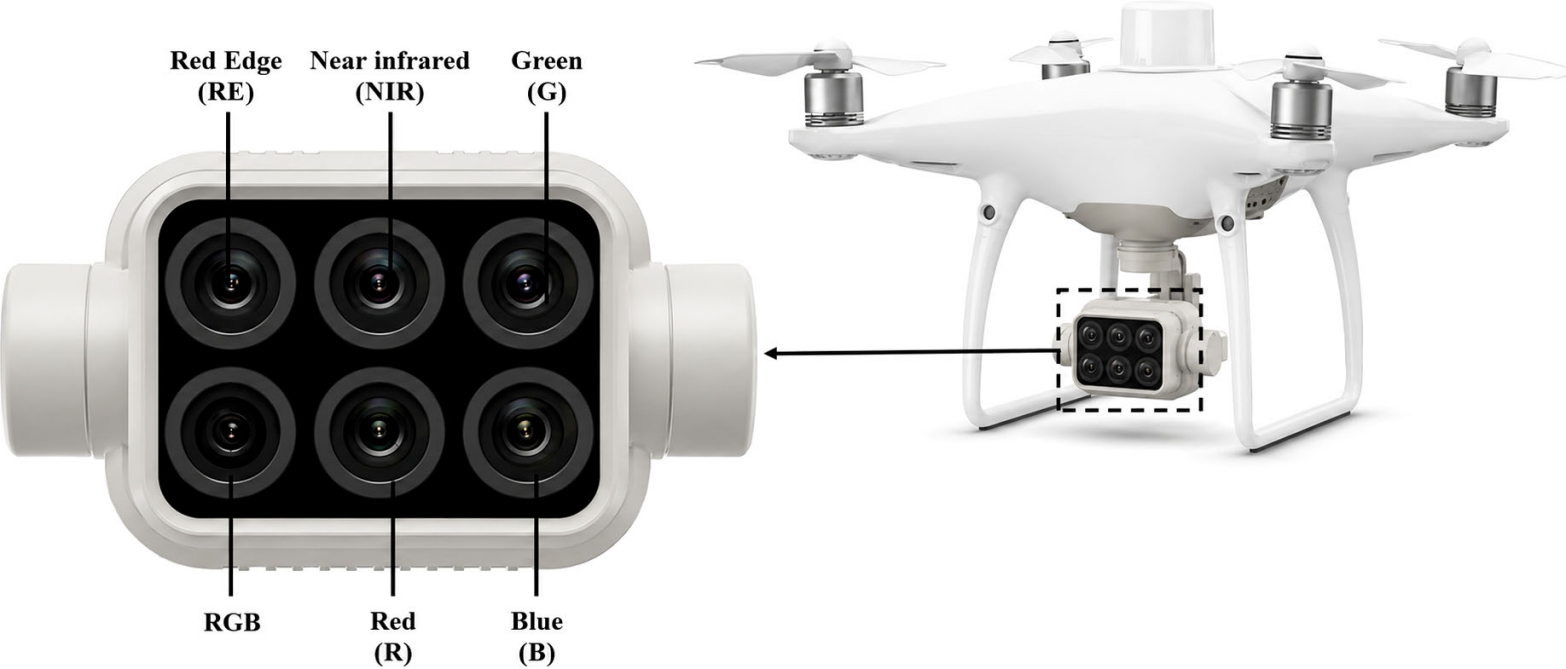
DJI Phantom 4 Multispectral (P4M) unmanned aerial vehicle platform used for multispectral image acquisition and DSM generation.

Multispectral imagery was simultaneously acquired using a DJI Phantom 4 Multispectral (P4M) UAV (DJI Innovations, Shenzhen, China). The platform is equipped with a high-precision multispectral imaging system comprising five spectral bands—blue, green, red, red-edge, and near-infrared—with central wavelengths of 450 nm, 560 nm, 650 nm, 730 nm, and 840 nm, respectively (**Figure 2**). The system can synchronously capture six images at a spatial resolution of 1600 × 1300 pixels, providing data support for crop phenotyping and digital surface model (DSM) construction.

UAV data acquisition was conducted on the same days as the corresponding ground LAI measurements. Flights were performed before destructive/manual LAI sampling to avoid canopy disturbance, and the ground measurements were completed immediately after the UAV flights within the same day. Data acquisition was conducted between 10:00 and 14:00 (Beijing time) under clear-sky conditions and low wind speeds (< 5 m s−1) to ensure sufficient illumination and optimal image quality. To guarantee spatial continuity and image sharpness, the flight parameters were set as follows: flight altitude of 30 m, flight speed of 10 m s−1, forward overlap of 80%, side overlap of 80%, ground sampling distance (GSD) of 0.8 cm pixel−1, and a camera gimbal pitch angle of −90.0°. A waypoint-based hovering scan was adopted, yielding a root mean square reprojection error of 0.84 pixels. The flight-line spacing (SP) was calculated as SP = W × (100 − side overlap)/100, and the number of flight lines (NFL) was determined as NFL = (survey width/SP) + 1. With an image swath width of 6.8 m (based on the P4M camera field of view), SP was calculated to be 1.36 m, resulting in 38 flight lines. Multispectral images were calibrated using a standard reflectance panel. Camera calibration was performed before each flight; radiometric correction was applied using the reflectance panel, and the panel digital number (DN) values were recorded for subsequent image radiometric correction.

The UAV-acquired multispectral images and the corresponding reflectance-panel images were imported into DJI Terra (v4.1.0; DJI, Shenzhen, Guangdong, China) for processing. The main procedures included image mosaicking, radiometric correction, and noise reduction. Orthomosaics for each multispectral band and the corresponding digital surface model (DSM) products were generated. Regions of interest (ROI) rasters for each experimental subplot were then created in ENVI Classic 5.5 (Harris Geospatial Solutions, Inc., Broomfield, CO, USA). Feature extraction algorithms for vegetation indices and DSM-derived metrics were implemented in Python 3.11 to generate the vegetation-index and DSM feature datasets. Based on previous studies, ten vegetation indices related to maize phenotypic traits were initially selected (**Table 1**) (Shu et al., 2023; Guo et al., 2024), from which the five indices with the highest correlations were retained for subsequent model development.

**Table 1:** Vegetation indices selected for this study and their calculation formulas.

Vegetation index	Equation	References
NDVI	$\text{NDVI}=\frac{\text{NIR}-\text{Red}}{\text{NIR}+\text{Red}}$	(Furlanetto et al., 2023)
MSAVI	$\text{MSAVI}=\frac{2*\text{NIR}+1-\sqrt{{\left(2*\text{NIR}+1\right)}^{2}-8*(\text{NIR}-\text{Red})}}{2}$	(Wu et al., 2007)
NDRE	$\text{NDRE}=\frac{\text{NIR}-\text{RedEdge}}{\text{NIR}+\text{RedEdge}}$	(Qiao et al., 2022)
GNDVI	$\text{GNDVI}=\frac{\text{NIR}-\text{Green}}{\text{NIR}+\text{Green}}$	(Kayad et al., 2022)
EVI	$\text{EVI}=2.5\frac{\text{NIR}-\text{Red}}{\text{NIR}+6\text{Red}-7.5\text{Blue}+1}$	(Shao et al., 2021)
OSAVI	$\text{OSAVI}=\frac{\text{NIR}-\text{Red}}{\text{NIR}+\text{Red}+0.16}$	(Liu et al., 2012)
RVI	$\text{RVI}=\frac{\text{NIR}}{\text{Red}}$	(Huang et al., 2023)
SAVI	$\text{SAVI}=1.5\frac{\text{NIR}-\text{Red}}{\text{NIR}+\text{Red}+0.5}$	(Campos et al., 2017)
GRVI	$\text{GRVI}=\frac{\text{Red}-\text{Green}}{\text{Red}+\text{Green}}$	(Sun et al., 2023)
IKAW	$\text{IKAW}=\frac{\text{Red}-\text{Blue}}{\text{Red}+\text{Blue}}$	(Kira et al., 2017)

Red, blue, green, NIR, and RedEdge represent reflectance in the red, blue, green, near-infrared, and red-edge bands, respectively.

#### 2.2.3 DSM feature extraction

In this study, DSM-derived features were extracted from the UAV-generated DSM products, including mean elevation (Mean) and four texture metrics derived from the gray-level co-occurrence matrix (GLCM) (Zhou et al., 2023): homogeneity (HOM), energy (ENE), contrast (CON), and entropy (ENT) (**Table 2**). The GLCM window size was set to 3×3 following the optimal-window validation reported in previous UAV texture studies (Liu et al., 2023b). Texture features were computed in four directions (0°, 45°, 90°, and 135°), and the final value of each texture metric was calculated as the mean across directions to reduce directional dependence. Z-score standardization was applied as Xnorm = (X − μ)/σ, where μ and σ are the mean and standard deviation of the corresponding feature. The extracted DSM-derived feature dataset was then used for feature screening and fused-feature construction, as described in Section 2.2.4.

**Table 2:** DSM-derived features selected for this study and their extraction formulas.

Texture Feature	Acronym	Formula	Description
Contrast	CON	$\sum_{\text{i}=0}^{\text{N}-1}\sum_{\text{j}=0}^{\text{N}-1}\text{P}\left(\text{i},\text{j}\right){\left(\text{i}-\text{j}\right)}^{2}$	Clarity of the texture
Homogeneity	HOM	$\sum_{\text{i}=0}^{\text{N}-1}\sum_{\text{j}=0}^{\text{N}-1}\text{P}\left(\text{i},\text{j}\right)\cdot \frac{1}{1+{\left(\text{i}-\text{j}\right)}^{2}}$	Homogeneity of the local texture
Energy	ENE	$\sqrt{\sum_{\text{i}=0}^{\text{N}-1}\sum_{\text{j}=0}^{\text{N}-1}\text{P}{\left(\text{i},\text{j}\right)}^{2}}$	Uniformity of the texture
Entropy	ENT	$\sum_{\text{i}=0}^{\text{N}-1}\sum_{\text{j}=0}^{\text{N}-1}\text{P}\left(\text{i},\text{j}\right)\cdot \left[-\ln\text{P}\left(\text{i},\text{j}\right)\right]$	Non-uniformity or complexity of the texture in the image

DSM, digital surface model; P(i,j), joint probability distribution of gray levels i and j in the gray-level co-occurrence matrix.

#### 2.2.4 Feature selection and predictor set construction

Feature selection was performed after the vegetation-index and DSM-derived feature datasets had been generated. Candidate VIs and DSM-derived metrics were screened using Pearson correlation analysis with measured LAI, and statistical significance was evaluated at P< 0.05 and P< 0.01. Variables were retained when they showed both predictive relevance and clear agronomic or structural interpretability. For the VI set, GNDVI, NDRE, GRVI, OSAVI, and IKAW were retained because they showed relatively strong LAI correlations and represented complementary spectral responses associated with canopy greenness, red-edge sensitivity, soil adjustment, and canopy water or structural sensitivity. For DSM-derived variables, the final three features were selected from mean elevation and four GLCM texture metrics according to their LAI correlation strength, significance level, and structural meaning. Highly correlated variables were not interpreted as independent causal factors; instead, their potential redundancy was considered during model interpretation and repeated validation. The correlation patterns and significance levels used to support feature selection are presented in Section 3.1.

### 2.3 Model development and evaluation

#### 2.3.1 Stacked ensemble learning model

**Figure 3:**
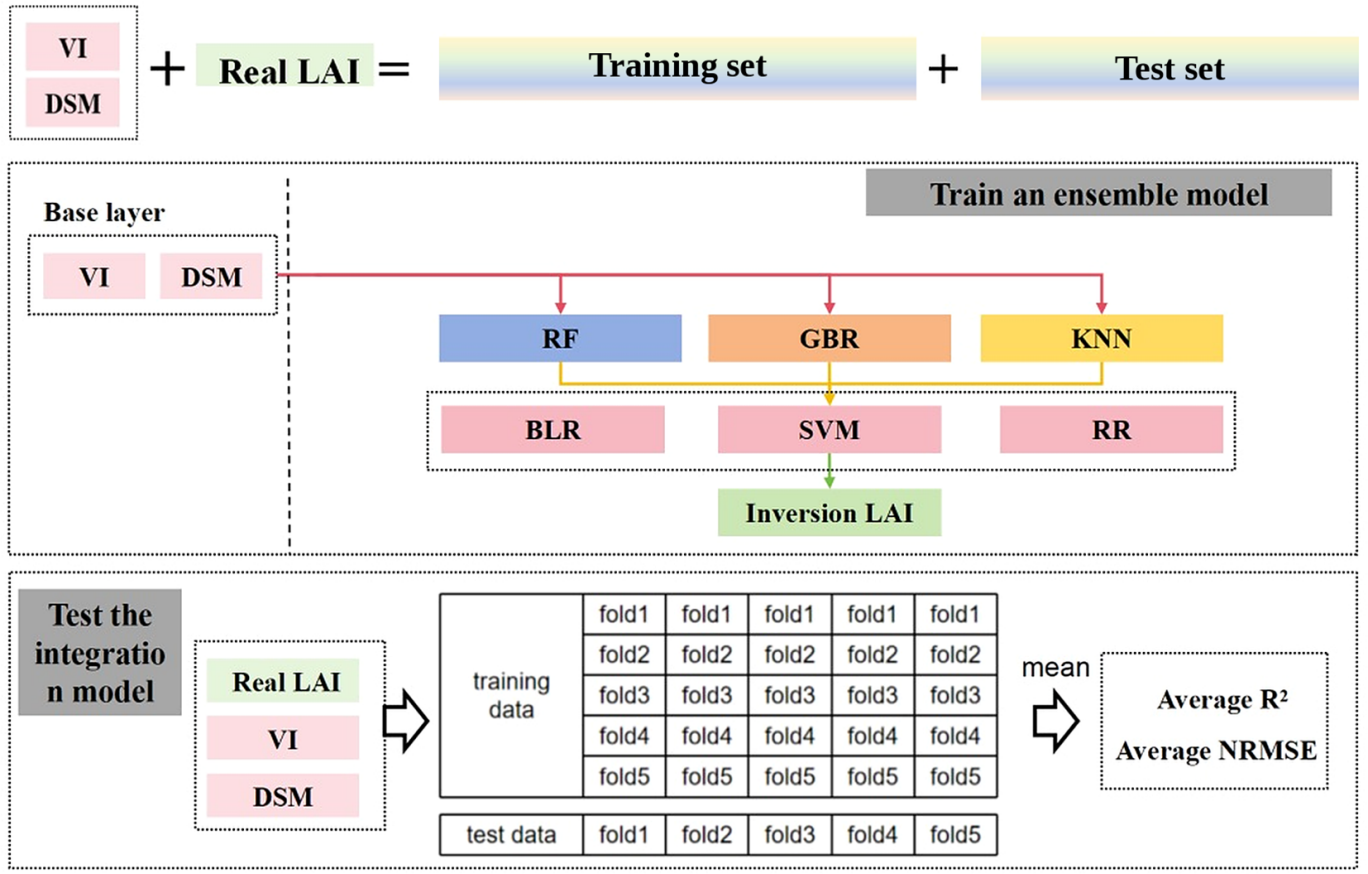
Schematic diagram of the stacking ensemble learning framework. RFR, random forest regressor; GBR, gradient boosting regressor; KNN, k-nearest neighbors; BLR, Bayesian linear regression; RR, ridge regression; SVM, support vector machine.

The stacking ensemble approach aims to improve predictive performance by combining multiple heterogeneous base learners. It adopts a multi-layer architecture: the first layer (base layer) consists of several base learners that generate preliminary predictions, while the second layer (meta-learning layer) trains a meta-learner on the outputs of the base learners to produce the final prediction. The model construction procedure of stacking is illustrated in **Figure 3**.

Specifically, the stacking ensemble in this study employed three complementary sub-models as base learners: a Random Forest Regressor (RFR), a Gradient Boosting Regressor (GBR), and k-Nearest Neighbors (KNN) (Zhao et al., 2024; Zhang et al., 2024). RFR and GBR are capable of capturing complex nonlinear relationships in the data, whereas KNN emphasizes local neighborhood characteristics. By stacking these learners, their complementary strengths can be integrated to improve prediction accuracy and stability. Bayesian Linear Regression (BLR), Ridge Regression (RR), and Support Vector Machine (SVM) were respectively used as meta-learners. BLR provides probabilistic interpretability and is well suited for handling noise in small-sample settings; RR mitigates multicollinearity via L2 regularization and is therefore compatible with multi-source feature fusion; and SVM, through kernel functions, can model nonlinear relationships and overcome the limitations of purely linear meta-models. These three meta-learners cover linear, nonlinear, and probabilistic prediction settings, helping to ensure the adaptability of the stacking framework across planting-density conditions. Accordingly, three stacking ensemble models were constructed using the three meta-learners, and the best-performing ensemble was selected. All models were implemented in Python 3.11 using the scikit-learn machine learning library.

#### 2.3.2 Dataset partitioning, validation strategy, and performance evaluation

The dataset was partitioned into a training set and a validation set using stratified sampling at a ratio of 4:1. Stratification was implemented by constructing a combined stratum label from planting density and growth stage. Specifically, each sample was assigned to one of nine density-stage strata: three density levels (57,000, 63,000, and 69,000 plants ha−1) × three growth stages (seedling, tasseling, and grain-filling). Within each stratum, samples were randomly shuffled and then split into training and validation subsets while preserving the original stratum proportions as closely as possible. This procedure ensured that both the training and validation sets contained representative samples from each planting-density and phenological-stage combination. Models were trained on the training set, and their performance was evaluated on the validation set. Before model fitting, continuous predictors were standardized using the parameters estimated from the training set and then applied to the validation set; this step was particularly important for distance- or kernel-based learners such as KNN and SVM. The coefficient of determination (R^2^) was used to quantify goodness of fit, and the normalized root mean square error (NRMSE) was reported in percentage form when presented in the Abstract and text. To more robustly assess model stability and generalization ability, model performance was further evaluated using repeated 10-fold cross-validation.

#### 2.3.3 Hyperparameter settings and tuning strategy

To ensure optimal and reproducible model performance, the hyperparameters of all base learners and meta-learners were tuned using grid search in conjunction with five-fold cross-validation (5-fold CV), with the objective function set to minimize the normalized root mean square error (NRMSE) (Ji et al., 2024; Li et al., 2024). The search ranges were defined with reference to prior studies on UAV-based LAI prediction in agricultural remote sensing (Zhai et al., 2023; Liu et al., 2012) and were further appropriately narrowed according to the feature dimensionality (eight features) and sample size (297 samples) of this study to reduce the risk of overfitting and avoid unnecessary computational redundancy (Zhao et al., 2025).

The specification of hyperparameter ranges and the determination of optimal values were closely aligned with the key characteristics of this study, namely a relatively small sample size (297 samples), a moderate feature dimensionality (eight features), and the multi-source feature-fusion setting. Accordingly, tuning was designed to balance model fitting capacity and generalization, control computational cost, and reduce the risk of overfitting. For the base learners, the number of trees and maximum tree depth in the RFR, as well as the number of neighbors in KNN, were selected to achieve adequate predictive power while avoiding redundant complexity; for the GBR, the learning rate and subsampling ratio were configured to support stable learning under small-sample conditions. For the meta-learners, the regularization strengths of BLR and RR were set to account for weak multicollinearity among the base-learner outputs, whereas the SVM adopted a radial basis function (RBF) kernel to capture nonlinear relationships among features. The penalty and kernel parameters were tuned to balance prediction accuracy, model complexity, and generalization ability, ensuring that the model was well adapted to the prediction scenario involving multi-source feature fusion.

The tuning procedure was implemented in Python 3.11 using the scikit-learn library. Specifically, the training set (80% of the full dataset) was split into five folds for cross-validation; in each iteration, one fold served as the validation set while the remaining folds were used for model training. For each algorithm, all combinations within the predefined hyperparameter grid were exhaustively evaluated. Models were trained for every combination, and the validation-set NRMSE was recorded. The hyperparameter combination yielding the minimum validation NRMSE was selected as the optimal setting. To ensure the stability of the optimal hyperparameters, the tuning process was repeated 50 times; the hyperparameter combination occurring most frequently across the 50 repetitions was adopted as the final configuration.

## 3 Results and analysis

### 3.1 Correlation analysis of features

**Figure 4:**
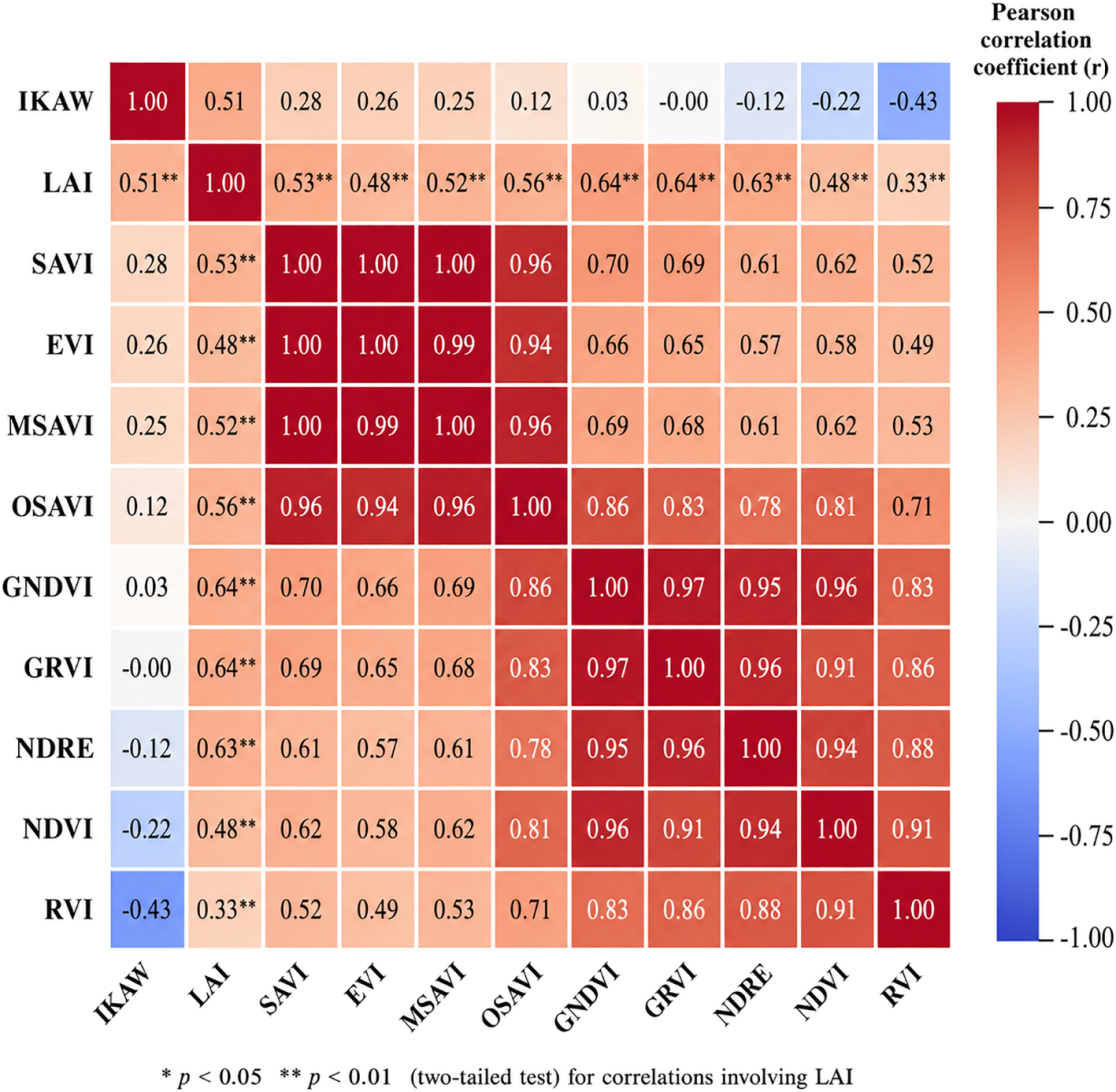
Pearson correlation heat map between vegetation indices and maize LAI. Values represent Pearson correlation coefficients (r); *P< 0.05 and **P< 0.01 indicate statistical significance. LAI, leaf area index; VI, vegetation index.

Correlation analysis between the vegetation indices and DSM-derived features and maize LAI showed that all ten vegetation indices were positively correlated with maize LAI (**Figure 4**).

**Figure 5:**
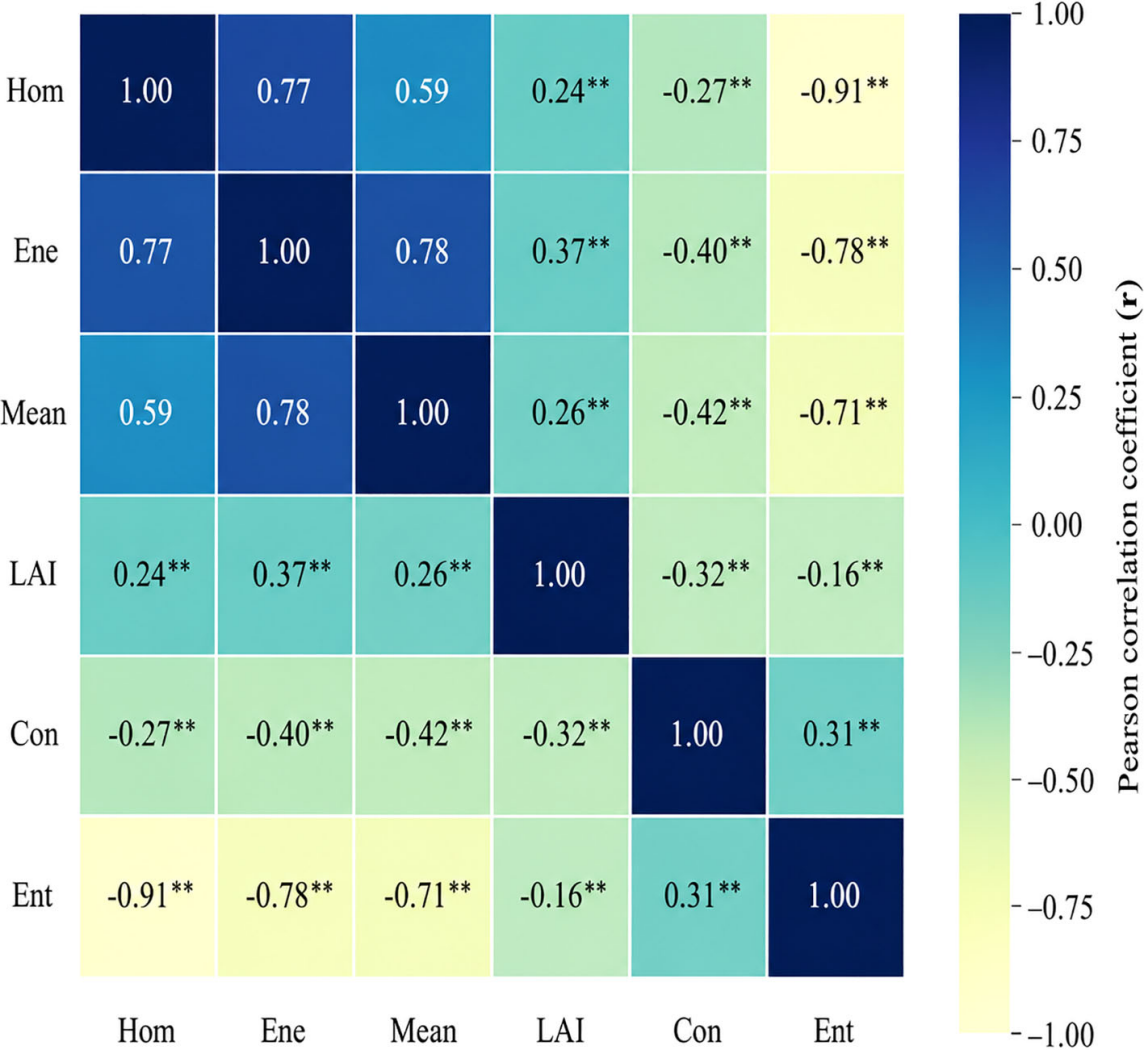
Pearson correlation heat map between DSM-derived features and maize LAI. Values represent Pearson correlation coefficients (r); **P < 0.01 indicates statistical significance for correlations involving LAI. DSM, digital surface model; HOM, homogeneity; ENE, energy; CON, contrast; ENT, entropy. The color scale represents correlation strength and direction.

Specifically, GNDVI, NDRE, GRVI, and OSAVI exhibited relatively strong correlations with LAI, indicating that these indices have clear advantages in characterizing maize canopy leaf growth. The correlations of GNDVI, NDRE, and GRVI were the most pronounced (r > 0.63; **P< 0.01). GNDVI reflects vegetation condition through the ratio of the green band to the near-infrared band; NDRE exploits the red-edge band, which is sensitive to chlorophyll concentration and internal leaf structure; and GRVI is sensitive to changes in vegetation cover and biomass. IKAW showed a moderate but significant correlation with LAI (r = 0.51; **P< 0.01) and relatively low correlations with several other VIs (r< 0.28), suggesting that it may provide complementary information. Therefore, GNDVI, NDRE, GRVI, OSAVI, and IKAW were retained as spectral predictors. For DSM-derived variables, five candidate features were initially considered: homogeneity (HOM), energy (ENE), mean, contrast (CON), and entropy (ENT). Pearson correlation analysis was used to evaluate both feature-LAI relationships and intercorrelations among predictors (**Figure 5**). The final DSM feature subset retained the variables with the strongest significant LAI associations and clear structural meaning. Because several selected spectral and texture predictors were intercorrelated, multicollinearity was considered during feature interpretation. The retained variables were used as predictive inputs rather than as independent causal factors. To reduce scale-related sensitivity in KNN and SVM, all predictors were standardized before modeling, and model robustness was evaluated through repeated cross-validation. Statistical significance is indicated in the correlation heatmaps as *P< 0.05 and **P< 0.01.

### 3.2 Comparative analysis of single-feature and fused-feature models

In this study, the predictive capability of single-feature and fused-feature inputs for maize LAI was evaluated by comparing the R^2^ and NRMSE of Random Forest (RF) models trained on different feature sets across planting-density conditions. RF was selected for this feature-level comparison because it is a robust nonlinear baseline that is widely used in UAV-based crop-parameter retrieval, can accommodate small to moderate sample sizes, and is relatively tolerant of correlated predictors. More importantly, using the same RF algorithm for VI-only, DSM-only, and VI + DSM inputs allowed the effect of feature fusion to be isolated from the effect of algorithm choice. Specifically, we compared models using single-source features—vegetation indices (VIs) and DSM-derived features—with models using fused features (VI + DSM) under different maize planting densities and summarized the mean prediction performance in **Table 3**.

**Table 3:** R^2^ and NRMSE values of RF models using different feature sets.

Feature set	Low density (57000plant ha−1)		Medium density (63000plant ha−1)		High density (69000plant ha−1)		All	
	R^2^	NRMSE	R^2^	NRMSE	R^2^	NRMSE	R^2^	NRMSE
VI+DSM-RF	0.892	0.077	0.924	0.063	0.860	0.088	0.892	0.076
DSM-RF	0.589	0.153	0.682	0.129	0.653	0.143	0.641	0.142
VI-RF	0.814	0.102	0.903	0.071	0.789	0.111	0.835	0.095

RF, random forest; VI, vegetation index; DSM, digital surface model; NRMSE, normalized root mean square error.

First, the predictive performance of single-feature models was evaluated. For models built using vegetation indices, the R^2^ values ranged from 0.789 to 0.903, indicating moderate to strong predictive capability. The highest R^2^ was obtained under the medium-density treatment (63,000 plants ha−1), reaching 0.903 with an NRMSE of 7.1%, suggesting that VIs alone can effectively capture density-related variability at this density level. In contrast, under high density, the R^2^ decreased to 0.789 and the NRMSE increased to 11.1%, indicating a slight degradation in VI-based prediction performance as planting density increased.

For models developed using DSM features, the R^2^ values were lower than those of the vegetation-index-based models. Specifically, R^2^ ranged from 0.589 to 0.682, with the highest value obtained under the medium-density treatment (63,000 plants ha−1) at 0.682 and an NRMSE of 12.9%. This indicates that DSM features alone were less effective than VIs for predicting LAI. In particular, the R^2^ values under low and high densities were 0.589 and 0.653, respectively. One plausible explanation is that at the medium planting density (63,000 plants ha−1), spectral information can already represent LAI reasonably well, and thus DSM-derived height information provides only limited additional gain. Moreover, the sensitivity of DSM texture features to canopy structure depends on image resolution; at low LAI, it is difficult to discriminate individual leaves from the soil background, which increases feature noise and degrades model performance.

After fusing vegetation indices with DSM features (VI + DSM), model performance for maize LAI prediction improved markedly under the same RF framework. The fused-feature RF model achieved R^2^ values ranging from 0.860 to 0.924 across planting densities. The highest R^2^ (0.924) and the lowest NRMSE (6.3%) were obtained under the medium-density treatment (63,000 plants ha−1). When pooling all densities, the fused-feature RF model achieved an overall R^2^ of 0.892 and an NRMSE of 7.6%, outperforming both VI-only and DSM-only RF models. These results indicate that DSM-derived structural/texture information mainly improved the feature representation of LAI, whereas the added value of stacking should be assessed separately under the same VI + DSM feature set.

### 3.3 Performance comparison of different ensemble models

**Figure 6:**
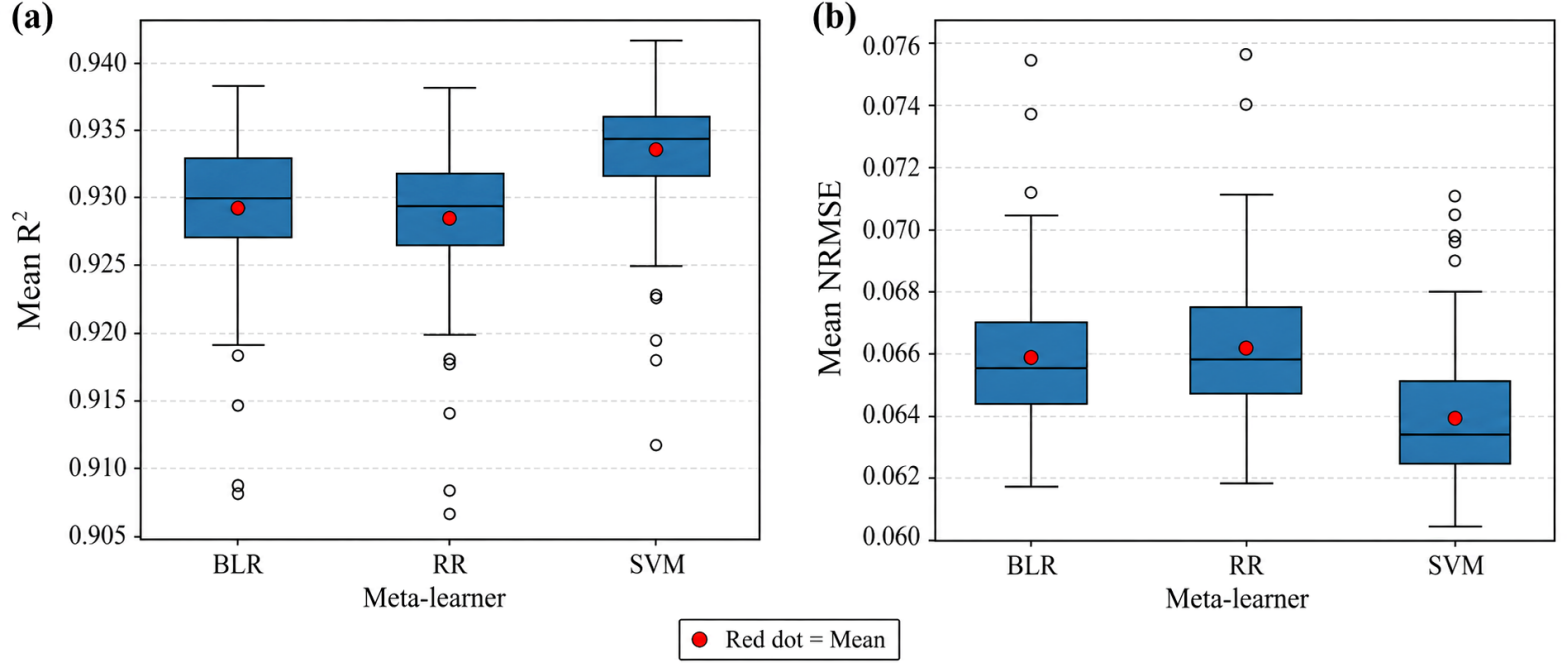
Performance comparison of stacking models constructed with three meta-learners based on 100 repeated trials. **(A)** R^2^ of the stacking models (dimensionless); **(B)** NRMSE of the stacking models (%). The x-axis indicates the meta-learner, and the legend identifies BLR, RR, and SVM. BLR, Bayesian linear regression; RR, ridge regression; SVM, support vector machine; NRMSE, normalized root mean square error.

To evaluate the performance of different meta-learners in the stacking ensemble, three stacking-based maize LAI prediction models were developed using the full growing-season dataset, with three algorithms respectively serving as the meta-learner. The evaluation metrics of LAI prediction are presented in **Figure 6**. The results show that the stacking ensemble with support vector regression (SVM) as the meta-learner achieved markedly higher mean predictive accuracy than the ensembles using the other meta-learners, with an average R^2^ of 0.934 and an average NRMSE of 0.064. In addition, the mean and median across repeated trials were the closest for the SVM-based stacking model, indicating the smallest influence of outliers in LAI prediction. This suggests that SVM is more suitable as the meta-learner for the stacking framework in this study.

**Figure 7:**
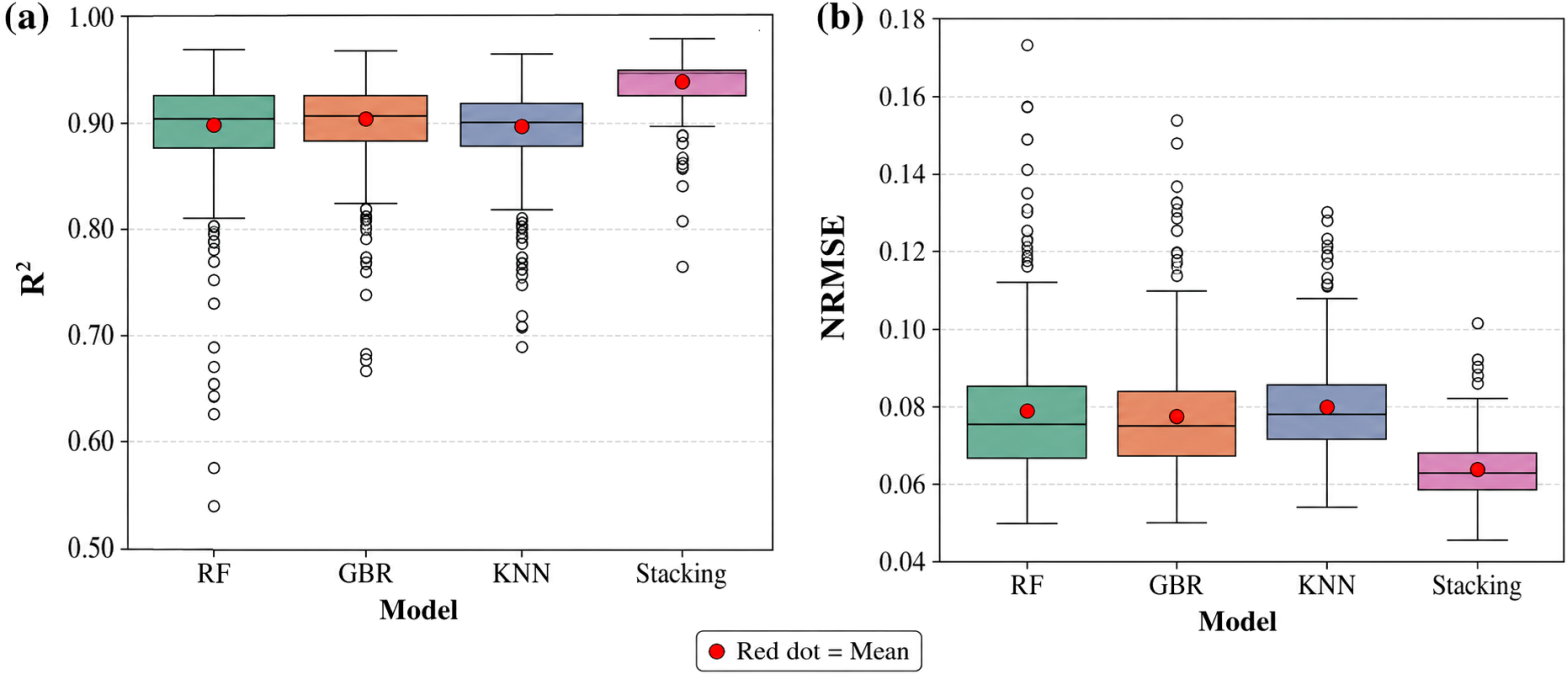
Performance comparison between the stacking ensemble model and individual base learners under the same VI + DSM feature set. **(A)** R^2^ values (dimensionless); **(B)** normalized root mean square error (NRMSE, %). The x-axis indicates model type, and the legend identifies individual learners and the stacking ensemble. VI, vegetation index; DSM, digital surface model.

Using the fused dataset combining DSM features and vegetation indices, we further compared maize LAI prediction models constructed with the stacking ensemble and with individual machine-learning algorithms (**Figure 7**). Among the single models, GBR achieved the best performance. The stacking ensemble produced the highest R^2^ and the lowest NRMSE among all models, although the improvement over the best individual learner was smaller than the improvement obtained by introducing DSM features into the VI-based RF model. These results indicate that stacking mainly provided an additional stability and accuracy gain under the same fused-feature setting by integrating the complementary tendencies of multiple base learners.

### 3.4 Comparison of model performance across planting densities

**Figure 8:**
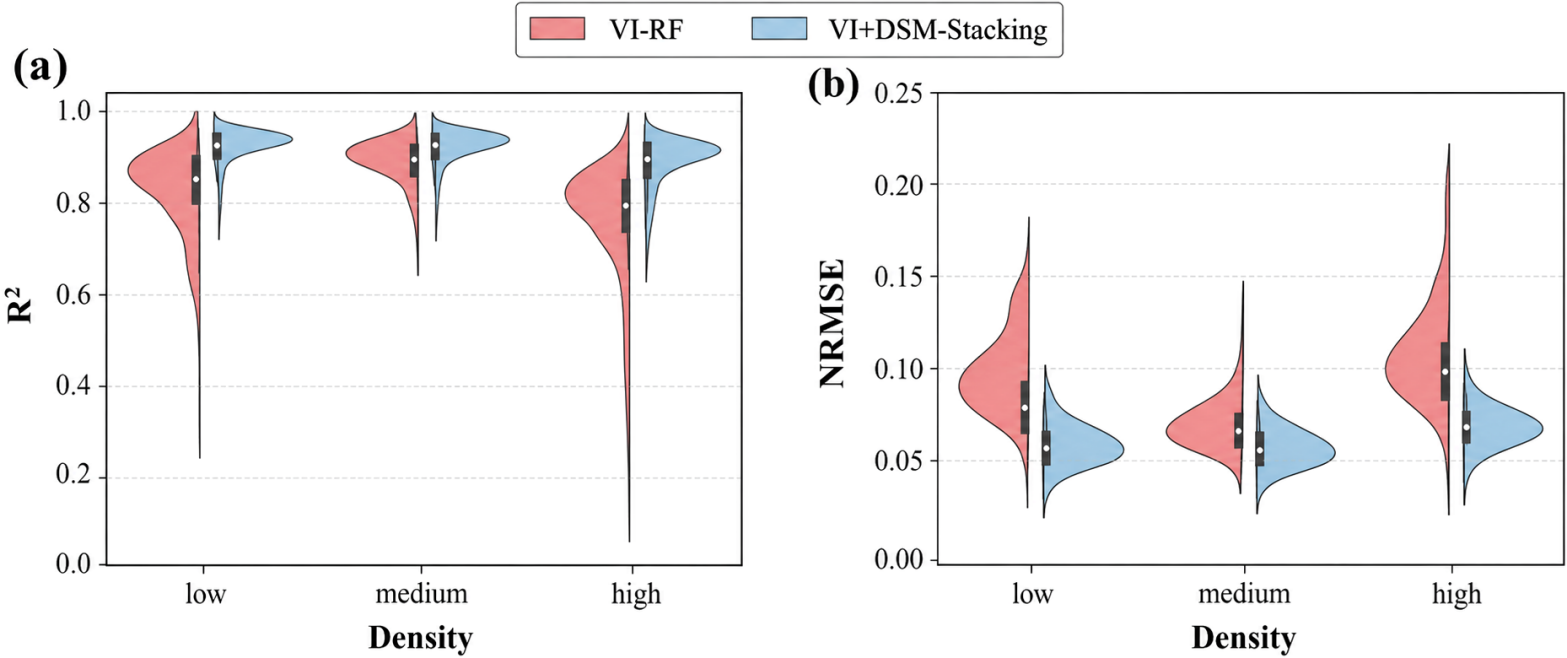
Endpoint performance comparison between the VI-RF baseline model and the final VI + DSM-Stacking workflow across planting densities based on 100 repeated trials. **(A)** R^2^ of the two models under low-, medium-, and high-density conditions; **(B)** NRMSE (%) of the two models under low-, medium-, and high-density conditions. The direct same-feature-set comparison between VI + DSM-RF and VI + DSM-Stacking is provided in **Table 4**. RF, random forest; VI, vegetation index; DSM, digital surface model; NRMSE, normalized root mean square error.

In this study, the coefficient of determination (R^2^) and normalized root mean square error (NRMSE) were compared among three models across planting densities: a vegetation-index random forest baseline (VI-RF), a fused-feature random forest model (VI + DSM-RF), and a fused-feature stacking model (VI + DSM-Stacking). This comparison was designed to separate two sources of improvement: the contribution of DSM feature fusion (VI-RF versus VI + DSM-RF) and the added value of stacking under the same fused-feature set (VI + DSM-RF versus VI + DSM-Stacking). **Table 4** provides the complete quantitative comparison among the three models. **Figure 8** was retained as an endpoint visualization showing the practical improvement from the spectral baseline model (VI-RF) to the final VI + DSM-Stacking workflow across planting-density conditions; the direct same-feature-set comparison between VI + DSM-RF and VI + DSM-Stacking should be interpreted from **Table 4**.

**Table 4:** R^2^ and NRMSE values of VI-RF, VI + DSM-RF, and VI + DSM-Stacking models.

Model	Low density (57000plant ha−1)		Medium density (63000plant ha−1)		High density (69000plant ha−1)		All	
	R^2^	NRMSE	R^2^	NRMSE	R^2^	NRMSE	R^2^	NRMSE
VI-RF	0.814	0.102	0.903	0.071	0.789	0.111	0.835	0.095
VI+DSM-RF	0.892	0.077	0.924	0.063	0.860	0.088	0.892	0.076
VI+DSM-Stacking	0.941	0.058	0.935	0.059	0.915	0.071	0.930	0.063

RF, random forest; VI, vegetation index; DSM, digital surface model; NRMSE, normalized root mean square error.

As shown in **Table 4**, feature fusion contributed the dominant improvement. Compared with VI-RF, the VI + DSM-RF model increased the overall R^2^ from 0.835 to 0.892 and reduced NRMSE from 0.095 to 0.076, indicating that DSM-derived structural information compensated for information gaps in the spectral predictors. Under the same VI + DSM feature set, the VI + DSM-Stacking model further improved the overall R^2^ from 0.892 to 0.930 and reduced NRMSE from 0.076 to 0.063. At the density-specific level, R^2^ increased from 0.892 to 0.941, from 0.924 to 0.935, and from 0.860 to 0.915 under low-, medium-, and high-density conditions, respectively, while NRMSE decreased from 0.077 to 0.058, from 0.063 to 0.059, and from 0.088 to 0.071. These results indicate that stacking provided a moderate but consistent incremental gain beyond VI + DSM-RF, rather than being the sole source of the total improvement over VI-RF.

The density-specific comparison further indicated that feature fusion and stacking were most beneficial under low- and high-density conditions. Under low density, sparse canopies increase soil background mixing, making single spectral features more susceptible to background interference. Under high density, canopy closure and leaf overlap induce spectral saturation and complicate radiative transfer, thereby reducing the discriminative ability of VIs. DSM texture and structural features can partially compensate for these two sources of error by describing canopy heterogeneity and spatial continuity. The repeated-trial distributions in **Figure 8** show the endpoint improvement from VI-RF to the final VI + DSM-Stacking workflow, whereas **Table 4** confirms the additional value of stacking under the same VI + DSM feature set. This revised interpretation avoids conflating feature-level and algorithm-level effects while retaining the practical comparison between the baseline model and the final workflow.

## 4 Discussion

Texture features can provide complementary information beyond spectral signals for LAI retrieval, and the gray-level co-occurrence matrix (GLCM) is among the most commonly used texture descriptors in UAV-based crop-parameter estimation. Previous studies have shown that jointly modeling GLCM texture with spectral features or vegetation indices can improve LAI estimation accuracy and alleviate saturation or underestimation under high-LAI conditions (Zou et al., 2024; Du et al., 2024b). Most existing studies have extracted texture directly from RGB or multispectral orthomosaics, whereas DSM-derived texture represents spatial variation in canopy surface height and roughness. This distinction is important because LAI is not only a spectral property but also an integrated canopy-structure variable. DSM/CHM products derived from UAV photogrammetry can characterize canopy height, surface continuity, and vertical heterogeneity, and their fusion with VIs has been reported to reduce instability in high-LAI ranges at certain growth stages (Zhang et al., 2022).

The physical mechanism by which DSM texture features mitigate soil background interference and spectral saturation can be interpreted from two aspects. Under low-density or early-growth conditions, exposed soil introduces mixed soil-canopy reflectance and weakens the monotonic relationship between VIs and LAI. DSM texture features, especially metrics related to homogeneity and energy, help distinguish continuous canopy surfaces from heterogeneous soil-plant mixtures because plant objects and soil background differ in height distribution and local surface roughness. Under high-density conditions, VIs may become less sensitive because of canopy closure, multiple scattering, leaf overlap, and reduced spectral contrast. In this situation, DSM-derived texture can still describe canopy surface roughness, spatial continuity, and height variability, thereby providing structural information that is not fully captured by spectral reflectance. In the present study, ENE, HOM, and mean were positively associated with LAI, whereas CON and ENT tended to decline as the canopy became more continuous. This pattern is consistent with the interpretation that increasing LAI increases canopy closure and reduces local grayscale or height-distribution heterogeneity in the DSM texture image.

Multi-source data fusion enables complementary information across feature dimensions, thereby compensating for the limitations of any single data source. Recent UAV-based LAI studies have increasingly emphasized the integration of spectral, texture, RGB, height, or DSM-derived structural features (Du et al., 2024; Zhao et al., 2025). Wang et al. (2022)showed that combining spectral and textural indices can alleviate saturation in forest LAI estimation, while Qiao et al. (2024) demonstrated that canopy morphological information can complement VIs in crop LAI monitoring. However, systematic evaluation of multispectral VIs combined with DSM-derived texture features under maize planting-density gradients remains limited. The present study addresses this gap by comparing VI-only, DSM-only, VI + DSM-RF, and VI + DSM-Stacking models and by separating feature-fusion effects from stacking-related effects.

Against this background, we compared maize LAI prediction performance using VI-based features, DSM-based features, and their fused set (VI + DSM), and further analyzed performance differences across planting-density conditions. The results indicate that the single VI-based model exhibited larger errors under low- and high-density conditions. Under low density, mixing of soil background and canopy signals reduced VI sensitivity to LAI, whereas under high density, enhanced canopy radiative interactions increased spectral complexity and led to saturation in some indices. After incorporating DSM features and fusing them with VIs, prediction accuracy under both low and high densities improved substantially (R^2^ increased by 9.58% and 8.99%, respectively, while NRMSE decreased by 24.51% and 20.72%, respectively), demonstrating that the structural information provided by DSM can compensate for VI limitations under extreme canopy-cover conditions. In contrast, the improvement under medium density was relatively modest (R^2^ increased by ~2.33% and NRMSE decreased by ~8.56%), likely because canopy cover at this density is moderate and VIs are less affected by background effects and saturation. Overall, VI–DSM fusion shows more pronounced advantages under low- and high-cover scenarios, thereby improving the reliability of LAI estimation; future work should further validate its transferability across additional years, cultivars, and fields.

Beyond feature-level fusion, algorithm-level ensemble learning can also enhance the accuracy and stability of LAI retrieval. In particular, stacking can integrate the outputs of multiple base learners through a meta-learner, thereby reducing the bias and variance of a single model. In UAV-based agronomic retrieval, ensemble strategies such as stacking have been demonstrated to improve the accuracy and robustness of estimating phenotypic parameters, including maize LAI, and to outperform individual models under multi-feature fusion settings (e.g., spectral + texture/structural features). In this study, introducing a stacking ensemble on top of the fused VI + DSM feature set yielded consistently better and more stable prediction performance across planting-density conditions, indicating that the complementarity among multiple learners can be effectively exploited.

Although this study improved the accuracy and stability of maize LAI estimation across planting-density conditions by combining VI-DSM fusion with a stacking ensemble framework, several limitations should be acknowledged. First, the experiment used a single maize cultivar (Beiyu 1521), and the model response may differ for cultivars with contrasting canopy architecture, leaf angle, plant height, or growth duration. Second, the UAV and ground observations were collected within one production season and one main production region in Dehong, Yunnan Province; therefore, interannual climate variability, soil background differences, and management heterogeneity were not fully represented. Third, because sowing date was partly confounded with field location and calendar time, this study did not interpret sowing date as an independent causal treatment in the final model analysis. Future experiments should adopt multi-year, multi-location, multi-cultivar, and fully crossed sowing-date × density designs to validate model transferability and to quantify genotype-by-environment-by-management interactions. Fourth, DSM-derived features are sensitive to photogrammetric reconstruction quality and ground reference conditions; integrating LiDAR-derived canopy height models or more robust 3D reconstruction workflows may further strengthen structural characterization. Finally, while stacking ensembles improved model performance, they also increased model complexity and computational cost. Future studies could improve deployment efficiency through feature selection, model compression, and coupling UAV-derived LAI with crop growth models for dynamic yield prediction.

## 5 Conclusions

This study developed and evaluated maize leaf area index (LAI) estimation models based on vegetation indices, DSM-derived features, VI-DSM feature fusion, and stacking ensemble learning under different planting-density conditions. The core innovation lies in combining UAV multispectral spectral information with DSM-derived structural and texture information and then using stacking ensemble learning to further improve prediction stability. The VI-RF model outperformed the DSM-only RF model (R^2^ = 0.835 and NRMSE = 9.5% versus R^2^ = 0.641 and NRMSE = 14.2%), confirming that spectral features remained the primary information source for maize LAI estimation. Under the same RF framework, fusing DSM features with VIs improved the overall R^2^ from 0.835 to 0.892 and reduced NRMSE from 9.5% to 7.6%, indicating that DSM-derived structural information compensated for spectral limitations, especially under sparse or dense canopy conditions. When the same VI + DSM feature set was used, the stacking model with SVM as the meta-learner further improved the overall R^2^ from 0.892 to 0.930 and reduced NRMSE from 7.6% to 6.3%. Thus, feature fusion contributed the dominant improvement, while stacking provided an additional but more moderate gain in accuracy and robustness. From the perspective of LAI remote-sensing estimation rather than yield optimization, the low-to-medium density range (57,000-63,000 plants ha−1) produced the most stable retrieval performance in this experiment; however, this range should not be interpreted as a universal agronomic recommendation without multi-year yield and management validation. Overall, the VI-DSM fusion plus stacking workflow provides a useful approach for UAV-based maize LAI monitoring across planting-density gradients, but further validation across years, sites, cultivars, and management conditions is needed before broad operational application.

## Data Availability

The raw data supporting the conclusions of this article will be made available by the authors, without undue reservation.

## References

[B1] Campos I. Neale C. M. U. Suyker A. E. Arkebauer T. J. Gonçalves I. Z. (2017). Reflectance-based crop coefficients REDUX: for operational evapotranspiration estimates in the age of high producing hybrid varieties. Agric. Water Manage. 187, 140–153. doi: 10.1016/j.agwat.2017.03.022. PMID: 38826717

[B2] Chen Z. Zhai W. Cheng Q. (2025). Enhancing maize LAI estimation accuracy using unmanned aerial vehicle remote sensing and deep learning techniques. Artif. Intell. Agric. 15, 482–495. doi: 10.1016/j.aiia.2025.04.008. PMID: 38826717

[B3] Dong T. Liu J. Shang J. Qian B. Ma B. Kovacs J. M. . (2019). Assessment of red-edge vegetation indices for crop leaf area index estimation. Remote Sens Environ. 222, 133–143. doi: 10.1016/j.rse.2018.12.032. PMID: 38826717

[B5] Du R. Lu J. Xiang Y. Zhang F. Chen J. Tang Z. . (2024a). Estimation of winter canola growth parameter from UAV multi-angular spectral-texture information using stacking-based ensemble learning model. Computers and Electronics in Agriculture. 222, 109074. doi: 10.1016/j.compag.2024.109074. PMID: 30654563

[B4] Du X. Zheng L. Zhu J. He Y . (2024b). Enhanced leaf area index estimation in rice by integrating UAV-based multi-source data. Remote Sensing. 16, 1138. doi: 10.3390/rs16071138. PMID: 38826717

[B7] Furlanetto J. Dal Ferro N. Longo M. Morari F. Chiozzotto R. (2023). LAI estimation through remotely sensed NDVI following hail defoliation in maize (Zea mays L.) using Sentinel-2 and UAV imagery. Precis Agric. 24, 1355–1379. doi: 10.1007/s11119-023-09993-9. PMID: 37363793 PMC9968646

[B8] Gao S. Zhong R. Yan K. Ma X. Chen X. Pu J. . (2023). Evaluating the saturation effect of vegetation indices in forests using 3D radiative transfer simulations and satellite observations. Remote Sens Environ. 295, 113665. doi: 10.1016/j.rse.2023.113665. PMID: 38826717

[B9] Guo Y. Hao F. Zhang X. He Y. Fu Y. H . (2024). Improving maize yield estimation by assimilating UAV-based LAI into WOFOST model. Field Crops Res. 315, 109477. doi: 10.1016/j.fcr.2024.109477. PMID: 38826717

[B10] Hirooka Y. Homma K. Shiraiwa T. (2018). Parameterization of the vertical distribution of leaf area index (LAI) in rice (Oryza sativa L.) using a plant canopy analyzer. Sci. Rep. 8, 6387. doi: 10.1038/s41598-018-24369-0. PMID: 29686403 PMC5913339

[B11] Huang X. Lin D. Mao X. Zhao Y . (2023). Multi-source data fusion for estimating maize leaf area index over the whole growing season under different mulching and irrigation conditions. Field Crops Res. 303, 109111. doi: 10.1016/j.fcr.2023.109111. PMID: 38826717

[B12] Ji Y. Liu Z. Cui Y. Liu R. Chen Z. Zong X. . (2024). Faba bean and pea harvest index estimations using aerial-based multimodal data and machine learning algorithms. Plant Physiol. 194, 1512–1526. doi: 10.1093/plphys/kiad577. PMID: 37935623 PMC10904323

[B13] Jin X. Ma J. Wen Z. Song K. (2015). Estimation of maize residue cover using Landsat-8 OLI image spectral information and textural features. Remote Sens 7, 14559–14575. doi: 10.3390/rs71114559. PMID: 30654563

[B14] Kayad A. Rodrigues F. A. Naranjo S. Plauborg F. (2022). Radiative transfer model inversion using high-resolution hyperspectral airborne imagery: retrieving maize LAI to assess biomass and grain yield. Field Crops Res. 282, 108449. doi: 10.1016/j.fcr.2022.108449. PMID: 35663617 PMC9025414

[B15] Kira O. Nguy-Robertson A. L. Arkebauer T. J. Linker R. Gitelson A. A. (2017). Toward generic models for green LAI estimation in maize and soybean: satellite observations. Remote Sens 9, 318. doi: 10.3390/rs9040318. PMID: 30654563

[B16] Li Y. Li C. Cheng Q. Chen L. Li Z. Zhai W. . (2024). Precision estimation of winter wheat crop height and above-ground biomass using unmanned aerial vehicle imagery and oblique photography point cloud data. Front. Plant Sci. 15, 1437350. doi: 10.3389/fpls.2024.1437350. PMID: 39359624 PMC11446220

[B17] Liu J. Pattey E. Jégo G. (2012). Assessment of vegetation indices for regional crop green LAI estimation from Landsat images over multiple growing seasons. Remote Sens Environ. 123, 347–358. doi: 10.1016/j.rse.2012.04.002. PMID: 38826717

[B18] Liu J. Zhu Y. Song L. Su X. Li J. Zheng J. . (2023b). Optimizing window size and directional parameters of GLCM texture features for estimating rice AGB based on UAVs multispectral imagery. Front. Plant Sci. 14, 1284235. doi: 10.3389/fpls.2023.1284235. PMID: 38192693 PMC10773816

[B19] Liu S. Jin X. Bai Y. Wu W. Cui N. Cheng M. . (2023a). UAV multispectral images for accurate estimation of the maize LAI considering the effect of soil background. Int. J. Appl. Earth Obs Geoinf 121, 103383. doi: 10.1016/j.jag.2023.103383. PMID: 38826717

[B20] Parker G. G. (2020). Tamm review: Leaf area index (LAI) is both a determinant and a consequence of important processes in vegetation canopies. For. Ecol. Manage. 477, 118496. doi: 10.1016/j.foreco.2020.118496. PMID: 38826717

[B21] Qiao D. Yang J. Bai B. Li G. Wang J . (2024). Non-destructive monitoring of peanut leaf area index by combing UAV spectral and textural characteristics. Remote Sens 16, 2182. doi: 10.3390/rs16122182. PMID: 30654563

[B22] Qiao L. Zhao R. Tang W. An L. Sun H. Li M. . (2022). Estimating maize LAI by exploring deep features of vegetation index map from UAV multispectral images. Field Crops Res. 289, 108739. doi: 10.1016/j.fcr.2022.108739. PMID: 38826717

[B23] Shao G. Han W. Zhang H. Liu S. Wang Y. Zhang L. . (2021). Mapping maize crop coefficient Kc using random forest algorithm based on leaf area index and UAV-based multispectral vegetation indices. Agric. Water Manage. 252, 106906. doi: 10.1016/j.agwat.2021.106906. PMID: 38826717

[B25] Shu M. Zhu J. Yang X. Gu X. Li B. Ma Y. (2023). A spectral decomposition method for estimating the leaf nitrogen status of maize by UAV-based hyperspectral imaging. Comput. Electron. Agric. 212, 108100. doi: 10.1016/j.compag.2023.108100. PMID: 38826717

[B26] Strachan I. B. McCaughey J. H. (1996). Spatial and vertical leaf area index of a deciduous forest resolved using the LAI-2000 Plant Canopy Analyzer. For. Sci. 42, 176–181. doi: 10.1093/forestscience/42.2.176

[B27] Sun X. Yang Z. Su P. Wei K. Wang Z. Yang C. . (2023). Non-destructive monitoring of maize LAI by fusing UAV spectral and textural features. Front. Plant Sci. 14, 1158837. doi: 10.3389/fpls.2023.1158837. PMID: 37063231 PMC10102429

[B28] Vincent G. Antin C. Laurans M. Heurtebize J. Durrieu S. Lavalley C. . (2017). Mapping plant area index of tropical evergreen forest by airborne laser scanning: a cross-validation study using LAI2200 optical sensor. Remote Sens Environ. 198, 254–266. doi: 10.1016/j.rse.2017.05.034. PMID: 38826717

[B30] Wang Q. Putri N. A. Gan Y. Song G . (2022). Combining both spectral and textural indices for alleviating saturation problem in forest LAI estimation using Sentinel-2 data. Geocarto Int. 37, 10511–10531. doi: 10.1080/10106049.2022.2037730. PMID: 37339054

[B29] Wang B. Zhu J. Sun S. Zhang L. Yan Y. Wang H. . (2026). Multimodal remote sensing combination for maize LAI estimation: stacking model development and phenology-specific feature sensitivity analysis. Artif. Intell. Agric. doi: 10.1016/j.aiia.2026.03.011. PMID: 38826717

[B31] Wu J. Wang D. Bauer M. E. (2007). Assessing broadband vegetation indices and QuickBird data in estimating leaf area index of corn and potato canopies. Field Crops Res. 102, 33–42. doi: 10.1016/j.fcr.2007.01.003. PMID: 38826717

[B32] Yang G. Liu J. Zhao C. Li Z. Huang Y. Yu H. . (2017). Unmanned aerial vehicle remote sensing for field-based crop phenotyping: current status and perspectives. Front. Plant Sci. 8, 1111. doi: 10.3389/fpls.2017.01111. PMID: 28713402 PMC5492853

[B35] Zhai W. Li C. Cheng Q. Ding F. Chen Z. (2023). Exploring multisource feature fusion and stacking ensemble learning for accurate estimation of maize chlorophyll content using unmanned aerial vehicle remote sensing. Remote Sens 15, 3454. doi: 10.3390/rs15133454. PMID: 30654563

[B38] Zhang P. Lu B. Shang J. Wang X. Hou Z. Jin S. . (2024). Ensemble learning for oat yield prediction using multi-growth stage UAV images. Remote Sens 16, 4575. doi: 10.3390/rs16234575. PMID: 30654563

[B36] Zhang X. Zhang K. Wu S. Shi H. Sun Y. Zhao Y. . (2022). An investigation of winter wheat leaf area index fitting model using spectral and canopy height model data from unmanned aerial vehicle imagery. Remote Sens 14, 5087. doi: 10.3390/rs14205087. PMID: 30654563

[B40] Zhao Z. Yao H. Zeng D. Jiang Z. Zhang X . (2025). UAV multi-source data fusion with super-resolution for accurate soybean leaf area index estimation. Front. Plant Sci. 16, 1700660. doi: 10.3389/fpls.2025.1700660. PMID: 41358364 PMC12675413

[B39] Zhao H. Wang J. Guo J. Hui X. Wang Y. Cai D. . (2024). Detecting water stress in winter wheat based on multifeature fusion from UAV remote sensing and stacking ensemble learning method. Remote Sens 16, 4100. doi: 10.3390/rs16214100. PMID: 30654563

[B41] Zhou H. Yang J. Lou W. Sheng L. Li D. Hu H. (2023). Improving grain yield prediction through fusion of multi-temporal spectral features and agronomic trait parameters derived from UAV imagery. Front. Plant Sci. 14, 1217448. doi: 10.3389/fpls.2023.1217448. PMID: 37908835 PMC10613988

[B42] Zou M. Liu Y. Fu M. Li C. Zhou Z. Meng H. . (2024). Combining spectral and texture feature of UAV image with plant height to improve LAI estimation of winter wheat at jointing stage. Front. Plant Sci. 14, 1272049. doi: 10.3389/fpls.2023.1272049. PMID: 38235191 PMC10791996

